# A systematic review and meta-analysis of genotype-based and individualized data analysis of *SLCO1B1* gene and statin-induced myopathy

**DOI:** 10.1038/s41397-021-00208-w

**Published:** 2021-02-19

**Authors:** Saowalak Turongkaravee, Jiraphun Jittikoon, Thitiya Lukkunaprasit, Sermsiri Sangroongruangsri, Usa Chaikledkaew, Ammarin Thakkinstian

**Affiliations:** 1 Social, Economic and Administrative Pharmacy (SEAP) Graduate Program, Faculty of Pharmacy, Mahidol University, Bangkok, Thailand; 2 Department of Biochemistry, Faculty of Pharmacy, Mahidol University, Bangkok, Thailand; 3 Department of Clinical Epidemiology and Biostatistics, Faculty of Medicine Ramathibodi Hospital, Mahidol University, Bangkok, Thailand; 4 Social and Administrative Pharmacy Division, Department of Pharmacy, Faculty of Pharmacy, Mahidol University, Bangkok, Thailand; 5 Mahidol University Health Technology Assessment (MUHTA) Graduate Program, Mahidol University, Bangkok, Thailand

**Keywords:** Genetic association study, Dyslipidaemias

## Abstract

This meta-analysis was conducted to determine the genotypic effects of rs4149056 and rs2306283 polymorphism in *SLCO1B1* gene on myopathy in patients with statin. Studies were searched using multiple databases and selected following inclusion criteria. Two reviewers independently performed data extraction and assessments for risk of bias. Fixed-or-random-effect was applied to pool allele frequency/effects. Mixed-effect logit model was used to pool genotypic effects using individual patient data. Heterogeneity and publication bias were explored. Fourteen studies were pooled for rs4149056; the minor C allele frequency were 15% in Caucasians and 14% in Asians. Six studies were pooled for rs2306283; the minor G allele frequency was 34% in Caucasian and 75% in Asians. Genotypic effects of rs4149056 polymorphism in Caucasians indicated that statin users who carried CC and TC genotypes had a significantly higher risk of myopathy than those who carried TT genotype, with a pooled odds ratio (OR) of 2.9 (95% confidence interval, 1.59, 5.34) and 1.6 (1.20, 2.16), respectively. For subgroup analysis, CC and TC genotypes also suggested a higher risk of myopathy in simvastatin users [OR = 2.8 (1.17, 6.77) and OR = 1.8 (1.15, 2.77), respectively] and in atorvastatin users [OR = 4.0 (1.23, 12.63) and OR = 2.0 (1.11, 3.52), respectively] than those who carried TT genotype. There was no significant association between rs2306283 polymorphism and myopathy in Caucasians and Asians. There was no evidence of publication bias for both polymorphisms.

## Introduction

Hypercholesterolemia is a significant risk factor for cardiovascular diseases (CVDs) which is a vital cause of death in the world [1]. The mortality rate of CVDs is likely to increase in developing countries, especially in Asia [2] where the age-adjusted mortality rate was 82–215 per 100,000 compared with 26–46 per 100,000 in Western countries [3]. Statins, the lipid-lowering drugs class, are the first-line treatments for hypercholesterolemia that were recommended by the American College of Cardiology/American Heart Association for the primary prevention of atherosclerotic cardiovascular disease in patients with low-density lipoprotein cholesterol (LDL-C) levels more than 190 mg/dl or diabetic patients [4]. Statins inhibit 3-hydroxy-3-metylglutaryl-CoA reductase (HMG-CoA reductase) which could reduce LDL-C levels up to 55% and decrease CVDs by 20–30% [5].

However, myopathy commonly occurred in statin users, leading to poor adherence [6, 7]. Its severity varied from myalgia toward life-threatening rhabdomyolysis [8]. The incidence of mild muscle pain, myopathy, and rhabdomyolysis was about 190, 11, and 3.4 per 100,000 patient-years [9], respectively. In Thailand, the Health Products Vigilance Center database of the Thai Food and Drug Administration from 2013 to 2017 reported that myopathy was listed in the top 20 adverse events caused by any statin [10]. The cause of myopathy is associated with receiving higher doses of statin or drug-interaction, which inhibit cytochrome P450 3A4 (i.e., gemfibrozil antifungals or macrolide antibiotics), or related to other risk factors (i.e., age, female, Asian descent, low body mass index, untreated hypothyroidism, or impaired renal/ hepatic function [6, 11, 12]). In addition, the genetic factors may play a role in statins-induced myopathy [13, 14]. Statin enters the systemic circulation through the influx transporter, the organic anion transporting polypeptide (OATP)1B1 encoded by the solute carrier organic anion transporter family member 1B1 gene (*SLCO1B1*) [15]. Two polymorphisms (rs2306283 and rs4149056) of *SLCO1B1* gene have been reportedly associated with statin and risk of myopathy in statin users, as it could influence the plasma concentration of statins [15–18]. Accordingly, clinical practice guidelines from the Clinical Pharmacogenetics Implementation Consortium and the Royal Dutch Association for the Advancement of Pharmacy-Pharmacogenetics Working Group (DPWG) recommend that patients who carry the minor C allele (TC or CC) of rs4149056 polymorphism may have an additional risk for simvastatin-induced myopathy. They should be prescribed a low dose of simvastatin (not exceeding 40 mg/day), or an alternative statin (e.g., pravastatin or rosuvastatin), and a routine creatine kinase (CK) surveillance should be considered [15, 19]. Besides, DPWG also recommends that patients with TC or CC genotype of rs4149056 polymorphism may have a higher risk of atorvastatin-related myopathy, therefore alternate agents should be considered [19]. In addition, rs2306283 polymorphism of *SLCO1B1* gene may potentially influence the pharmacokinetics and pharmacodynamics of statins [17, 20–22], because all statins are substrates of OATP1B1 transporter. Therefore, the effect of rs4149056 and rs2306283 polymorphism and myopathy in any statins should be investigated.

Recently, five meta-analyses (MA) [23–27], which included individual studies upto 2017, investigated the association between *SLCO1B1* gene and myopathy in statin users. They mainly focused on the *SLCO1B1* (rs4149056) polymorphisms; only one MA also considered the rs2306283 polymorphisms in Caucasians [23]. However, these MAs did not include Asian studies and they pooled only minor allele effects or genetic model (i.e., recessive, co-dominant, dominant) rather than genotypic effects of each polymorphism in three genotypes (i.e., aa, Aa, and AA). Additionally, frequencies are different between the regions or population. For example, rs4149056 variant is the highest frequency in the native Americans and Europeans, whereas rs2306283 variant is the highest frequency in the Southeast Asian population [28, 29] which could affect the risk of myopathy in this population.

Therefore, this MA was conducted to assess the effect of these two polymorphisms of *SLCO1B1* gene on myopathy in both Caucasian and Asian patients who received statin. In addition, a subgroup analysis was performed by statin types. Moreover, we also estimated both allele frequencies and mode of genetic effects of each polymorphism separately by ethnicity, as suggested by the guidelines of the Human Genome Epidemiology Network [30].

## Materials and methods

This systematic review protocol was registered with PROSPERO, the International prospective register of systematic reviews (identification number CRD42018105282 and available from: http://www.crd.york.ac.uk/PROSPERO/display_record.php?ID=CRD42018105282). This review was conducted according to the guidelines of Preferred Reporting Items for Systematic Reviews and Meta-Analysis [31] and recently published guidance from the Human Genome Epidemiology Network for reporting gene-disease associations [30].

### Identification of studies

The studies were located from the MEDLINE via PubMed and Scopus databases up to April 2019, based on search terms of gene (i.e., rs2306283 or rs4149056 within the *SLCO1B1* genes) and outcome (i.e., myopathy) domains, see Supplementary Table S1. Searching was updated every 3 months, and the reference lists of relevant studies were also explored.

### Selection of studies

Two reviewers (ST and SS) independently selected studies by screening titles and abstracts. If a decision for eligibility could be made, the full articles were reviewed. Any types of studies were included if they met all of the following criteria: (1) studied in general adults (aged 18 years or older) who received statin regardless indications, (2) studied two polymorphisms (i.e., rs2306283 or rs4149056) of the *SLCO1B1* gene, and (3) had myopathy as the outcome. The studies were excluded if their data were insufficient for pooling and authors did not provide additional data after being contacted twice, as well as non-English studies. Any disagreements were resolved through consensus with the third author (TL).

### Outcome of interest

The primary outcome was myopathy defined by muscle weakness after statin use with/without confirming by CK higher rising three times, compared to the upper-normal limit [32].

### Data extraction

Data were extracted independently by two reviewers (ST and SS) focusing on five parts, i.e., general information, study characteristics, patient characteristics, genes and outcome, and data for pooling, see Table 1. If summary data were not reported, summary statistic data (i.e., odds ratio (OR)) were extracted. Authors were contacted if their reported data were insufficient for pooling. Disagreements were resolved by consensus with the third author (TL).

**Table 1:** General characteristics of included studies.

Study	Author	Year	Country	Ethnicity	Study design	SNP	Statin types	Duration on statin therapy	Population disease	Mean age in case/control, year (SD)	% Males in case/control	Type of case
1	Bai et al. [47]	2018	China	Asian	Cohort	rs2306283 rs4149056	Rosuvastatin	6 months	Coronary artery disease	61 (10.93)/63 (10.33)	71/76	Muscle symptom and CK elevation >4 times ULN
2	Willrich et al. [38]	2018	USA	Caucasian	Case-control	rs2306283 rs4149056	Various statins	N/A	Hyperlipidemia	66 (9.4)/66 (10.2)	57/57	Muscle symptom or elevation of CK activity^a^
3	Bakar et al. [39]	2017	UK	Caucasian	Cohort	rs4149056	Various statins Simvastatin (86%)	6 months	Cardiovascular disease	60 (1.1)/61 (0.58)	42/56	Muscle symptom and CK elevation >4–10 times ULN^b^
4	Liu et al. [49]	2017	China	Asian	Case-control	rs2306283	Various statins	6 months	Coronary artery disease	61 (10.81)/63 (10.72)	82/83	Muscle symptom or CK elevation >10 times ULN
5	Khine et al. [46]	2016	USA	Mixed ethnicity	Cohort	rs4149056	Various statins	3 months	Hypercholesterolemia	57 (3.5)/54 (4)	65/57	Muscle symptoms
6	Sai et al. [48]	2016	Japan	Asian	Case-control	rs4149056	Various statins	N/A	Patients with statin therapy	68 (9.9)/N/A	58/N/A	Muscle symptom with CK elevation >3–10 times ULN
7	Mirošević Skvrce et al. [40]	2015	Croatia	Caucasian	Case-control	rs2306283 rs4149056	Atorvastatin	N/A	Patients with atorvastatin therapy	56 (4.5)/60 (4.25)	50/50	Muscle symptom or CK elevation >4–10 times ULN
8	Ferrari et al. [41]	2014	Italy	Caucasian	Case-control	rs2306283 rs4149056	Various statins	N/A	Patients with statin therapy	62 (9.9)/61 (9.9)	39/39	CK elevation >3 times ULN
9	Carr et al. [26]	2013	UK	Caucasian	Case-control	rs4149056	Various statins	12 months	Patients with statin therapy	70 (10.4)/71 (8.7)	71/64	CK elevation >4 times ULN
10	Brunham et al. [42]	2012	The Netherlands	Caucasian	Case-control	rs4149056	Various statins	N/A	Patients with statin therapy	53 (13)/57 (12)	78/73	CK elevation >10 times ULN
11	Donnelly et al. [17]	2011	UK	Caucasian	Cohort	rs2306283 rs4149056	Various statins (simvastatin 61%)	12 months	Patients with statin therapy	64 (10.6)/N/A	51/N/A	CK elevation >1–3 times ULN
12	Marciante et al. [43]	2011	USA	Caucasian	Case-control	rs4149056	Cerevastatin	N/A	Patients with cerevastatin therapy	64 (10.6)/74 (4.1)	39/45	Muscle pain or weakness with CK elevation >10 times ULN
13	Linde et al. [44]	2010	USA	Caucasian	Cohort	rs4149056	Various statins	N/A	Patients with statin therapy	59 (10)/59 (13.8)	54/64	Muscle pain or weakness
14	Voora et al. [45]	2009	USA	Caucasian	RCT	rs4149056	Various statins	4 months	Hyperlipidemia	58 (10)/56 (11)	33/50	Muscle symptom or CK elevation >3 times ULN
15	Link et al. [18]	2008	UK	Caucasian	RCT	rs4149056	Simvastatin	12 months	Myocardial infarction	67 (9)/N/A	73/83	Muscle symptom and CK elevation >3–10 times ULN

*CK* creatine kinase, *N/A* not available, *RCT* randomized controlled trial, *SD* standard deviation, *SNP* single nucleotide polymorphism, *ULN* upper limit of normal.

^a^Reference range for CK: males: 0.884–885.712 μkat/l; females: 0.646–642.992 μkat/l.

^b^Reference ranges for CK: Male: 10–190 ULN; female 10–160 ULN.

### Risk of bias assessment

Two reviewers (ST and TL) independently assessed a risk of bias for genetic association studies [33] considering five domains: selection bias, information bias, confounding bias, multiple tests and selective outcome report, and Hardy–Weinberg equilibrium (HWE) assessment. Each item was classified as low/no (“yes”), possible/high (“no”), or “unclear” risk of bias. For any disagreement, this was resolved by the third author (SS).

### Statistical analysis

Statistical analysis was performed following the method for meta-analysis of genetic association studies [34]. HWE was assessed in the control groups to detect equilibrium of two alleles (A and a, with frequencies p and q, respectively) by using the Chi-square (*χ*^2^) or exact test goodness-of-fit where appropriate. The studies were not included in pooling if they did not comply with HWE. Heterogeneity and its degree were assessed using Cochrane’s *Q* test and *I*^2^ statistic. If the heterogeneity was present (*p* value  < 0.1 or *I*^2^ ≥ 25%), the sources of heterogeneity were explored by meta-regression or subgroup analysis by potential factors (i.e., age groups, percent female, statin type, and duration use).

A minor allele frequency (MAF) in the control groups was pooled and stratified by ethnicity. Gene effects were estimated using two approaches (i.e., per allele and per genotype approaches). For the per allele approach, ORs of minor allele a vs major allele A along with 95% confidence interval (CI) were estimated. The ORs were pooled using a random-effect model by DerSimonian and Laird method [35] if heterogeneity was present (*p* value  < 0.1 or *I*^2^ ≥ 25%); otherwise, a fixed-effect model with inverse variance method was applied. For the per genotype approach, the OR_1_ (aa vs AA) and OR_2_ (Aa vs AA) were estimated using a mixed-effect logistic regression.

A sensitivity analysis was determined by including and excluding studies that did not comply with HWE in the main pooling to see robustness of the results based on a method for meta-analysis of genetic association studies [30, 34]. Lastly, publication bias was assessed using a funnel plot and the Egger’s test [36]. A contour enhanced-funnel plot was constructed if any asymmetry was suggested by funnel plot or Egger’s test [37]. All analyses were performed using STATA software version 15.0 (StataCorp, College Station, Texas, USA). A *p* value  < 0.05 was considered as statistically significant.

## Results

### Identifying of included studies

**Fig. 1:**
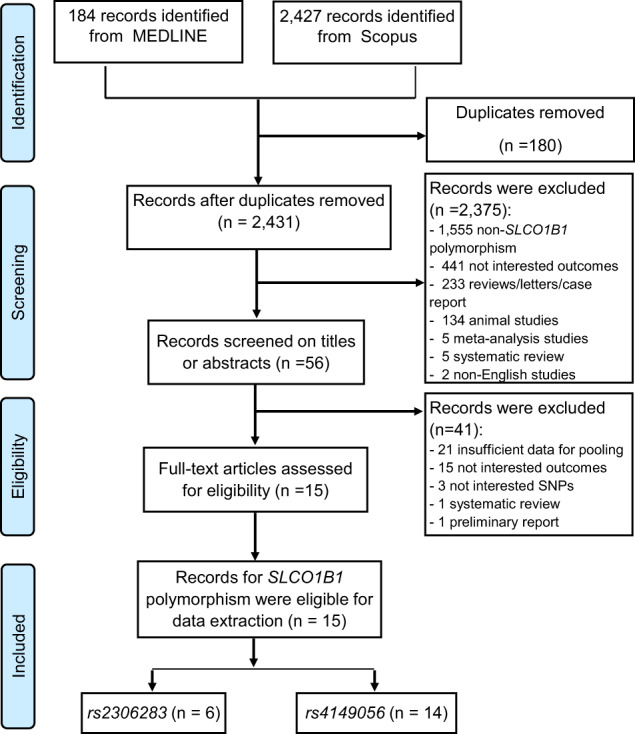
PRISMA flow diagram of identifying and selecting studies for a systematic review and meta-analysis.

A total of 2427 and 184 studies were, respectively, located from Scopus and MEDLINE via PubMed, but only 15 studies were eligible, see Fig. 1. Fourteen and six studies assessed effects of rs4149056 [17, 18, 26, 38–48] and rs2306283 polymorphisms [17, 38, 40, 41, 47, 49].

The characteristics of 15 included studies were demonstrated in Table 1. Of them, eight, five, and two were case-controls [26, 38, 40–43, 48, 49], cohorts [17, 39, 44, 46, 47], and randomized controlled trials [18, 45]. Eleven, three, and one studies were conducted in Caucasians [17, 18, 26, 38–45], Asians [47–49], and mixed populations [46]. Eleven studies were investigated in patients receiving various statins [17, 26, 38, 39, 41, 42, 44–46, 48, 49], the rests were simvastatin [18], atorvastatin [40], cerivastatin [43], and rosuvastatin only [47]. Mean age ranged from 53 to 74 years, and the proportion of males ranged from 33% to 83%. The type of case was defined by muscle symptoms with/without CK confirmation.

### Risk of bias assessment

The results of bias assessment are shown in Supplementary Table S2. All studies were clearly defined for diagnosis of myopathy and controls (non-myopathy). Therefore, they were low risk. However, nine studies (60%) were unclear in quality control of genotyping. One study was unclear about the balance of ethnicity between case and control, which might be prone to population stratification.

Moreover, the risk of confounding bias might be presented in two studies that did not mention adjusting confounders. All studies demonstrated a significant association between SNPs and myopathy as a result of low risk of bias from selective outcome reporting. Ten of 15 studies (67%) assessed HWE.

### Pooling results

#### rs4149056 polymorphism

Fourteen studies investigated the association between rs4149056 and myopathy. Of these, 11 studies [17, 18, 26, 38–45] and two studies [47, 48] were conducted in Caucasians and Asians, respectively, while only one study investigated mixed ethnicity [46]. Ten studies investigated various statins with subgroup of statin types, i.e., atorvastatin, simvastatin, cerivastatin, and rosuvastatin. Allele frequencies across myopathy and control groups were described (Supplementary Table S3). All studies were compiled with the HWE rule. The MAF C among 11 Caucasian studies was higher in myopathy than that in control patients with the pooled frequencies (95% CI) of 0.26 (0.21, 0.32) and 0.15 (0.14, 0.16), respectively. The allele effect was highly heterogeneous (*χ*^2^ = 59.53, df = 10, *p* = < 0.001, *I*^2^ = 83.2%) with the pooled OR (C vs T) of 1.84 (1.35, 2.53). Therefore, a random-effect model was used to pool ORs across studies.

The pooled frequency of C allele in two Asian studies was 0.24 (0.18, 0.30) and 0.14 (0.14, 0.15) in case and control groups, respectively. However, the allele effect was homogeneous (*χ*^2^ = 0.73, df = 1, *p* = 0.392, *I*^2^ = 0%), with the pooled ORs (95% CI) of C vs T of 1.87 (1.34, 2.60). A fixed-effect model was used to pool ORs across studies. The results suggested that statin users who carried minor C allele had ~1.8 times higher risk of myopathy than those who carried T allele in Caucasians and Asians. Sensitivity analysis was not performed, because all included studies were complied with HWE (which suggested that there was no indication of disequilibrium for all studies) and were included in the main pooling.

**Fig. 2:**
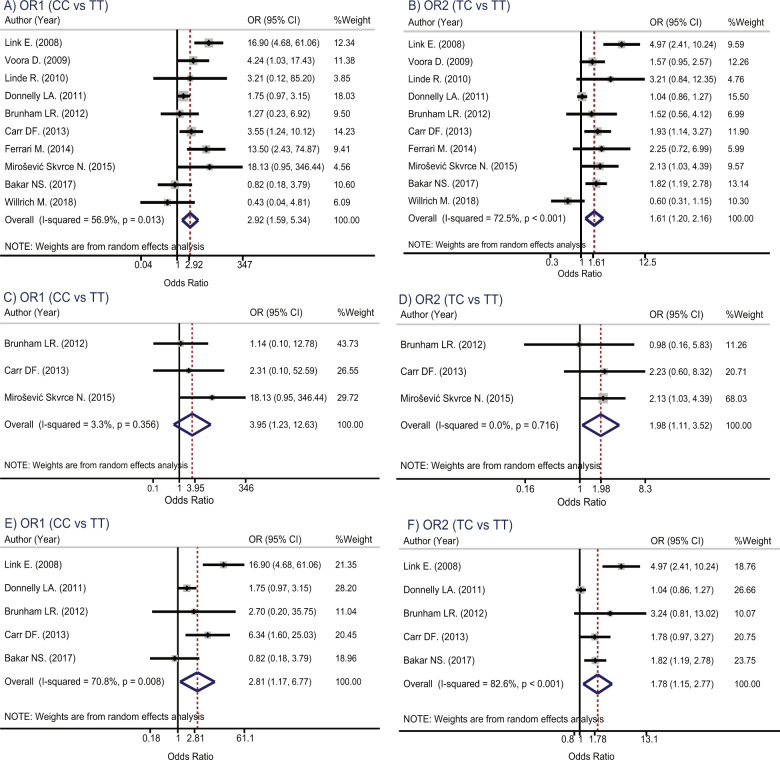
Forest plots for pooling genotypic effects of *SLCO1B1* gene rs4149056 polymorphisms in Caucasians. **A** OR_1_ (CC vs TT) in statin users, **B** OR_2_ (TC vs TT) in statin users, **C** OR_1_ (CC vs TT) in atorvastatin users, **D** OR_2_ (TC vs TT) in atorvastatin users, **E** OR_1_ (CC vs TT) in simvastatin users, and **F** OR_2_ (TC vs TT) in simvastatin users.

Genotype effects were estimated separately by ethnicity, see Table 2. For rs4149056 polymorphism, genotype effects were sufficient for pooling only in ten out of Caucasians studies [17, 18, 26, 38–42, 44, 45] (*n* = 1433 vs 2878 for myopathy vs control groups). Heterogeneity was present for both OR_1_ (CC vs TT: *χ*^2^ = 20.9, df = 9, *p* = 0.013, *I*^2^ = 56.9%) and OR_2_ (TC vs TT: *χ*^2^ = 32.7, df = 9, *p* < 0.001, *I*^2^ = 72.5%). A mixed-effect logit model was used to pool OR_1_ and OR_2_ across studies, which yielded OR_1_ and OR_2_ of 2.9 (1.59, 5.34) and 1.6 (1.20, 2.16), respectively (Fig. 2A, B), indicating that statin users who carried CC and TC genotypes had 2.9 and 1.6 times significantly higher risk of myopathy than those statin users who carried TT genotype.

**Table 2:** Pooled genotypic effects of *SLCO1B1* gene rs4149056 polymorphisms in Caucasian with specific type of statin.

Study	Author	Year	Myopathy					Control					CC vs TT		TC vs TT	
			No. of subjects in case	Genotype				No. of subjects in control	Genotype				OR1	95% CI	OR2	95% CI
				TT		TC	CC		TT		TC	CC				
**Caucasian with statin users**																
1	Willrich et al. [38]	2018	89	67		21	1	89	57		30	2	0.43	0.04, 4.81	0.60	0.31, 1.15
2	Bakar et al. [39]	2017	125	78		45	2	476	353		112	11	0.82	0.18, 3.79	1.82	1.19, 2.78
3	Mirošević Skvrce et al. [40]	2015	60	34		22	4	90	69		21	0	18.13	0.95, 346.44	2.13	1.03, 4.39
4	Ferrari et al. [41]	2014	33	8		13	12	33	18		13	2	13.50	2.43,74.87	2.25	0.72, 6.99
5	Carr et al. [26]	2013	76	40		30	6	372	260		101	11	3.55	1.24, 10.12	1.93	1.14, 3.27
6	Brunham et al. [42]	2012	25	15		8	2	83	57		20	6	1.27	0.23, 6.92	1.52	0.56, 4.12
7	Donnelly et al. [17]	2011	816	565		227	24	1275	905		348	22	1.75	0.97, 3.15	1.04	0.86, 1.27
8	Linde et al. [44]	2010	27	14		12	1	19	15		4	0	3.21	0.12, 85.20	3.21	0.84, 12.35
9	Voora et al. [45]	2009	97	62		31	4	351	263		84	4	4.24	1.03, 17.43	1.57	0.95, 2.57
10	Link et al. [18]	2008	85	29		35	21	90	70		17	3	16.9	4.68, 61.06	4.97	2.41, 10.24
	**Pooled**												**2.92**	**(1.59, 5.34)**	**1.61**	**(1.20, 2.16)**
**Caucasian with atorvastatin users**																
1	Mirošević Skvrce et al. [40]	2015	60	34	22		4	90	69	21		0	18.13	0.95, 346.44	2.13	1.03, 4.39
2	Carr et al. [26]	2013	11	7	4		0	110	86	22		2	2.31	0.10, 52.59	2.23	0.60, 8.32
3	Brunham et al. [42]	2012	10	7	2		1	34	24	7		3	1.14	0.10, 12.78	0.98	0.16, 5.83
	**Pooled**												**3.95**	**(1.23, 12.63)**	**1.98**	**(1.11, 3.52)**
**Caucasian with simvastatin users**																
1	Bakar et al. [39]	2017	125	78	45		2	476	353	112		11	0.82	0.18, 3.79	1.82	1.19, 2.78
2	Carr et al. [26]	2013	59	29	25		5	222	147	71		4	6.34	1.60, 25.03	1.78	0.97, 3.27
3	Brunham et al. [42]	2012	12	5	6		1	39	27	10		2	2.70	0.20, 35.75	3.24	0.81, 13.02
4	Donnelly et al. [17]	2011	816	565	227		24	1275	905	348		22	1.75	0.97, 3.15	1.04	0.86, 1.27
5	Link et al. [18]	2008	85	29	35		21	90	70	17		3	16.90	4.68, 61.06	4.97	2.41, 10.24
	**Pooled**												**2.81**	**(1.17, 6.77)**	**1.78**	**(1.15, 2.77)**

*CI* confidence interval, *OR* odds ratio.

The potential sources of heterogeneity were explored by fitting age, female gender, duration of therapy, and the statins type in a meta-regression model (see Supplementary Table S6), indicating that statin type might be the source of heterogeneity. A subgroup analysis was performed accordingly. Genotype effects in atorvastatin users were homogeneous with the pooled OR_1_ and OR_2_ of 4.0 (1.23, 12.63) and 2.0 (1.11, 3.52), see Fig. 2C, D; whereas effects in simvastatin users were still heterogeneous with these corresponding ORs of 2.8 (1.17, 6.77) and 1.8 (1.15, 2.77), see Fig. 2E, F. Publication bias was assessed by funnel plot and Egger’s test for pooled OR_1_ and OR_2_ in Caucasian with statin users, indicating no evidence of asymmetry of funnels for both ORs, see Supplementary Table S7 and Fig. S1.

#### rs2306283 polymorphism

Four and two studies assessed the association between rs2306283 and myopathy in Caucasians and Asians, and all studies complied with HWE. The pooled frequency of minor G allele in controls was a bit lower in Caucasians than that in Asians, i.e., 0.34 (0.27, 0.42) and 0.75 (0.74, 0.77), respectively. The ORs (G vs A) were 1.00 (0.71, 1.43) in Caucasians and 1.17 (0.84, 1.64) in Asians, see Supplementary Table S4. Sensitivity analysis was not performed, because all included studies were complied with HWE and were included in the main pooling.

**Fig. 3:**
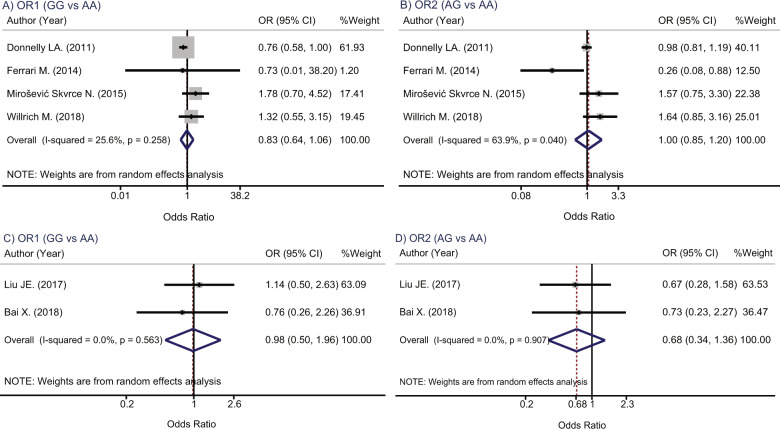
Forest plots for pooling genotypic effects of *SLCO1B1* gene rs2306283 polymorphisms in statin users. **A** OR_1_ (GG vs AA) in Caucasians, **B** OR_2_ (AG vs AA) in Caucasians, **C** OR_1_ (GG vs AA) in Asians, **D** OR_2_ (AG vs AA) in Asians.

Genotype effects were pooled among four Caucasians studies (*n* = 998 vs 1487 for myopathy vs control groups) and two Asian studies (*n* = 199 vs 956 for myopathy vs control groups), see Supplementary Table S5. The pooled OR_1_ (GG vs AA) and OR_2_ (AG vs AA) in statin users among Caucasians were 0.8 (0.64, 1.06) and 1.0 (0.85, 1.20) with moderate heterogeneity *I*^2^ of 25.6% and *I*^2^ = 63.9%, respectively (Fig. 3A, B). These corresponding ORs in statin users among Asians were 0.98 (0.50, 1.96) and 0.68 (0.34, 1.36) with the *I*^2^ of 0.0% for both ORs (Fig. 3C, D).

Because the number of included studies was only four and two studies in Caucasians and Asians, respectively, the publication bias and meta-regression were not performed as the power of detecting was low.

## Discussion

This meta-analysis of genetic association studies was conducted to assess the genetic effects of single nucleotide polymorphisms (rs4149056 and rs2306283) of the *SLCO1B1* gene on myopathy. Accordingly, the *SLCO1B1* gene is located on chromosome 12p12.1 (Online Mendelian Inheritance in Man # 604843) [50]. Two polymorphisms (rs2306283 and rs4149056) of *SLCO1B1* gene have been reported that they could influence statin and the risk of myopathy association [15–18]. Because the *SLCO1B1* gene is able to induce variations in plasma concentrations of statin through the influx transporter OATP1B1, it could influence the pharmacokinetic and pharmacodynamic profiles of statins [14, 51, 52].

Fourteen studies were included for rs4149056, and six studies for rs2306283 in the main pooling. The frequency of the minor allele for each polymorphism was pooled and the magnitude of genotype effects was determined by ethnicity. The analysis was performed to include additional studies provided from previous evidence and complied with the guidelines of the Human Genome Epidemiology Network [30]. The finding suggested that the minor C allele frequencies for rs4149056 polymorphism in Caucasian were slightly higher than that in Asian ethnicities, yielding a frequency of 15% and 14%, respectively. Indeed, the previous reviews reported that the C allele was associated with reduction of transport activity, which resulted in decreasing hepatic uptake and thus increasing plasma concentration of statins and a higher risk of myopathy. However, the effect is varied on the different types and dose of statins [16, 51, 53, 54]. Likewise, our study contributes to robust associations in Caucasians, suggesting that individuals who were taking various types of statin therapies and carried the minor C allele, which were CC or TC genotypes of rs4149056 polymorphism, may have higher risks of myopathy by 2.9 and 1.6 times, respectively, than those who carried TT genotype. The results were consistent with previous MA [23–27], although there were different study designs with our study using mixed-effect logit model with individual patient data for pooling genotypic effects. Accordingly, we confirmed previous findings by including extensively more studies and demonstrated the genotype effects in three genotypes (i.e., aa, Aa, and AA), rather than assuming a specific genetic model (i.e., recessive, co-dominant, dominant) to avoid misleading estimates of the OR when an inappropriate model is assumed [36]. For subgroup analysis, which was stratified by statin types, the results suggested that individuals receiving specific types of statin (i.e., simvastatin and atorvastatin) had significant effect estimates similar to various statin among CC or TC genotypes carriers. Simvastatin users who were carrying CC or TC genotypes of rs4149056 polymorphism had increased risks of myopathy by ~2.8 and 1.8 times, compared with those carrying the TT genotype. These findings were consistent with previous genome-wide association studies, which uncovered that carriers of CC and TC of rs4149056 had 16.9- and 4.5-times higher risk of myopathy than those of TT in users of simvastatin 80 mg/daily [18]. However, our study did not perform a specific dosage of simvastatin because of insufficient data for pooling from original studies and authors did not provide additional data after being contacted twice. Furthermore, the risk of myopathy was higher in atorvastatin users by ~4.0 and 2.0 times among CC or TC genotypes carriers as compared with those who carried the TT genotype.

Our findings further supported the role of the *SLCO1B1* genotype in statin-associated myopathy and suggested that this association may be more robust for atorvastatin and simvastatin. There were some reasons suggesting that individuals who received atorvastatin may have higher risk of myopathy than those who took simvastatin. It may explain why patients who obtained higher equivalent doses of statins or more potent statins, in terms of lipid-lowering ability, may have an increasing tendency for myopathy risk. Consequently, included studies on atorvastatin used dosage between 20 and 80 mg daily, which was classified as a moderate to high-intensity therapy. On the other hand, included studies on simvastatin mostly used 10–40 mg daily, which was a low to moderate-intensity therapy. Nevertheless, we did not report a specific dosage for any statin because of insufficient data for pooling from the original studies.

In addition, we performed TC + CC vs TT of rs4149056 polymorphism among ten studies in Caucasians. The results showed that individuals who carried TC + CC genotype also had a higher risk of myopathy when compared with TT genotype in statin users, with a pooled OR of 1.9 (95% CI: 1.28, 2.83; *p* < 0.001), and simvastatin users 2.2 (1.2, 4.2; *p* < 0.001) and atorvastatin users 2.1 (1.2, 3.8; *p* = 0.585). Our finding is similar to Xiang et al. [27]. The results suggested that patients who carrying the minor C allele (i.e., TC, CC or TC + CC) may have an additional risk of myopathy when compared with TT genotype. However, we included additional studies provided from previous evidence and there were different study designs with our study using mixed-effect logit model with individual patient data for pooling.

For the rs2306283, our study showed that the minor G allele frequencies were common in Asians, but rare in Caucasians, yielding a frequency of 75% in Asians and 34% in Caucasians. Nevertheless, this study suggested that there was no significant association between rs2306283 polymorphism and myopathy in Asians and Caucasians with statin users. It would be probably caused by the small numbers of included studies. Furthermore, the role of rs2306283 polymorphism in the transporter function was still controversial. For instance, the G allele might increase plasma concentration and cause myotoxicity [55]. On the other hand, it might increase transporter activity resulting in the lower oral bioavailability or decreased plasma concentration of statin and pravastatin [14, 56, 57], while the latter could be an attenuation of lower risk of statin-induced myopathy in patients who carried GG or AG genotypes [17]. Therefore, we would need more association studies in both Caucasian and Asian populations to confirm our findings.

### Strengths and limitations of the study

Our study had several strengths. Data analysis was performed following a standard method for meta-analysis of genetic association studies [30, 34]. We also pooled allele frequency and genetic effects separately, which was a requirement of the Human Genome Epidemiology Network [30]. Moreover, genotype effects were pooled using a genetic model-free approach with more studies in both rs4149056 and rs2306283 polymorphisms, compared with previous evidence, especially in the Asian population. Furthermore, the gene effects were estimated from individual patient data of genotypes and outcome of individual studies. Our study applied a mixed-effect logistic regression and separately by ethnicity to fit genotypes. The finding could be generalized as compared with previous studies because it is the first meta-analysis that included Asian studies given that the *SLCO1B1* gene is generally distributed and different between regions [28].

Moreover, the included studies had a definite diagnosis of myopathy case and control groups and all studies complied with HWE in the main pooling as a result of limiting potential bias in outcome measures. Indeed, HWE is a mathematical equation that can be used to calculate the genetic variation of a population at equilibrium as well as provides a quality of genetic association studies in terms of design and conduct. The deviation from HWE in the control group may be due to selection bias, population stratification and genotyping errors, which may bias the estimates of genetic effects [58–60].

Besides, our study reported no evidence of publication bias due to small study effects. Moreover, sources of heterogeneity were explored. However, other characteristics may cause heterogeneity, because either studies did not report from original studies (i.e., co-medication, a dosage of administration, hypothyroidism, chronic kidney disease, excess alcohol intake) or the data were not sufficient for pooling.

Given that all types of statins were substrates of OATP1B1 transporter, we would need more genetic association studies of the *SLCO1B1* gene and myopathy among other types of statin to confirm that the effects could be a class effect or specific types of statin only. Future research including analysis cost-effectiveness study on the *SLCO1B1* gene is also required. It may be useful for assessing the value of money of pharmacogenetic testing before prescribing statin, preventing the adverse events, and improving patient’s adherence considering that pharmacogenetic testing plays an extensive role on precision and personalized medicine.

### Policy recommendation

This study provided an understanding of the roles of genetic and drug response. Our findings may be considered an extension of traditional approaches to treat a disease, which is useful for physicians, as they could select a drug treatment or intervention based on a patient’s molecular profile. This could lead to a reduction of harmful adverse effects, ensuring an effective outcome as well as containing costs from a “trial-and-error” approach to disease treatment. Moreover, policy-makers could make evidence-based decisions for the selection of drugs or alternative drugs on a list of Thai National List of Essential Medicines for patients through the genetic information added to the clinical and economic evidence.

## Conclusions

In summary, this meta-analysis provides a robust association in Caucasians indicating that individuals who carried the minor C allele, with CC or TC genotypes of rs4149056 polymorphism may particularly have higher risks of myopathy among various statin users and especially in those who received simvastatin and atorvastatin, compared with those who carried the TT genotype. No significant association between rs2306283 polymorphism and myopathy was caused by various statin users was found in Asian and Caucasian populations.

## Supplementary information

Supplementary information
