# Supplementary material for: A systematic review and meta-analysis of genotype-based and individualized data analysis of SLCO1B1 gene and statin-induced myopathy
Source: Pharmacogenomics J. 2021 Feb 19;21(3):296–307. doi: 10.1038/s41397-021-00208-w (PMC8159730; doi:10.1038/s41397-021-00208-w)
Supplement: Supplementary file 1 — Supplementary information [file 41397_2021_208_MOESM1_ESM.pdf]

Supplementary information

Table S1. Search strategies A) Search strategies in MEDLINE via PubMed. B) Search strategies in Scopus.

Table S2. Risk of bias assessment

Table S3. Allele frequencies of major and minor allele for *SLCO1B1* gene rs4149056 polymorphisms by ethnicity

Table S4. Allele frequencies of major and minor allele for *SLCO1B1* gene rs2306283 polymorphisms by ethnicity

Table S5. Pooled genotypic effects of *SLCO1B1* gene rs2306283 polymorphisms in Caucasian and Asian with statin users.

Table S6. Exploring the source of heterogeneity and subgroup analysis of *SLCO1B1* gene rs4149056 polymorphisms in Caucasians

Table S7. Egger's tests for *SLCO1B1* gene rs4149056 polymorphisms in Caucasians

Figure S1. Funnel plots of publication bias of *SLCO1B1* gene rs4149056 polymorphisms in Caucasian with statin users.

Figure S2. Funnel plots of publication bias of *SLCO1B1* gene rs4149056 polymorphisms in Caucasian with atorvasstatin users.

Figure S3. Funnel plots of publication bias of *SLCO1B1* gene rs4149056 polymorphisms in Caucasian with simvastatin users.

Figure S4. Funnel plots of publication bias of *SLCO1B1* gene rs2306283 polymorphisms in Caucasian with statin users.

Figure S5. Funnel plots of publication bias of *SLCO1B1* gene rs2306283 polymorphisms in Asian with statin users.

Table S1. Search strategies

A) Search strategies in MEDLINE via PubMed.

**Domain(s)**	**Search**	**Query**	**Items found**
Interventions and outcomes	#54	#35 AND #53	**184**
All terms for Muscle symptom outcome	#53	#46 OR #50	217,008
	#52	#50 OR #51	37,377
	#51	Search (("Creatine Kinase"[Mesh])	25,309
	#50	#47 OR #48 OR #49	37,377
	#49	Search "Creatine Phosphotransferase"	69
	#48	Search "Creatine Phosphokinase"	4,852
	#47	Search "Creatine Kinase"	35,212
	#46	#38 OR #40 OR #44 OR #45	186,864
	#45	Search Myositis	21,016
	#44	Search Myalgia	7,047
	#43	#41 OR #42	8,950
	#42	Search "Rhabdomyolysis"[Mesh]	6,128
	#41	#39 OR #40	8,950
	#40	Search Rhabdomyolyses	8,950

Domain(s)	Search	Query	Items found
	#39	Search Rhabdomyolysis	8,946
	#38	#36 OR #37	178,720
	#37	Search Myopathies	172,373
	#36	Search Myopathy	177,367
Interventions	#35	#16 AND #34	816
	#34	#20 OR #26 OR #30 OR #33	1,362
	#33	#31 OR #32	727
	#32	Search "organic anion transporter 1B1"	19
	#31	Search OATP1B1	716
	#30	#27 OR #28 OR #29	755
	#29	Search "OATP-C"	86
	#28	Search "OATP-C Transport Protein"	712
	#27	Search "Organic Anion Transport Polypeptide C"	2
	#26	#23 OR #24 OR #25	750
	#25	Search "LST-1"	34
	#24	Search "LST-1 Transport Protein"	712
	#23	Search "Liver-Specific Organic Anion Transporter"	17

**Domain(s)**	**Search**	**Query**	**Items found**
	#22	#20 OR #21	1,040
	#21	Search "Solute Carrier Organic Anion Transporter Family Member 1b1"[Mesh]	695
	#20	#18 OR #19	1,040
	#19	Search SLCO1B1	830
	#18	Search "Solute Carrier Organic Anion Transporter 1b1"	830
	#17	#3 OR #6 OR #11 OR #14 OR #15	2,717,259
	#16	#1 OR #5 OR #9 OR #14 OR #15	2,717,259
	#15	Search allele	238,268
	#14	#12 OR #13	324,000
	#13	Search polymorphisms	301,499
	#12	Search polymorphism	297,391
	#11	#9 OR #10	**133,943 133,943**
	#10	Search "single nucleotide polymorphism"	106,781
	#9	#7 OR #8	133,943
	#8	Search SNPs	123,408
	#7	Search SNP	50,490
	#6	#4 OR #5	466,001

Domain(s)	Search	Query	Items found
	#5	Search genotypes	466,001
	#4	Search genotype	437,142
	#3	#1 OR #2	2,464,779
	#2	Search genes	1,351,565
	#1	Search gene	2,464,779

B) Search strategies in Scopus

Domain(s)	Search	Query	Items found
Interventions and outcomes	#44	#29 AND #43	**2,427**
All terms for Muscle symptom outcome	#43	#38 OR #42	273,392
	#42	#39 OR #40 OR #41	99,297
	#41	Search "Creatine Phosphotransferase"	977
	#40	Search "Creatine Phosphokinase"	14,727
	#39	Search "Creatine Kinase"	94,054
	#38	#30 OR #35 OR #36 OR #37	192,320
	#37	Search Myositis	38,318
	#36	Search Myalgia	50,622
	#35	#33 OR #34	31,028
	#34	Search Rhabdomyolyses	192
	#33	Search Rhabdomyolysis	31,012
	#32	#30 OR #31	101,394
	#31	Search Myopathies	101,394
	#30	Search Myopathy	101,394
Interventions	#29	#15 AND #28	4,496

Domain(s)	Search	Query	Items found
	#28	#18 OR #21 OR #24 OR #27	6,793
	#27	#25 OR #26	3,925
	#26	Search "organic anion transporter 1B1"	888
	#25	Search OATP1B1	3,615
	#24	#22 OR #23	1,715
	#23	Search "OATP-C"	1,684
	#22	Search "Organic Anion Transport Polypeptide C"	80
	#21	#19 OR #20	914
	#20	Search "LST-1"	658
	#19	Search " Liver-Specific Organic Anion Transporter"	826
	#18	#16 OR #17	3,778
	#17	Search SLCO1B1	3,551
	#16	Search "Solute Carrier Organic Anion Transporter 1b1"	673
	#15	#2 OR #5 OR #10 OR #11 OR #14	6,673,889
	#14	Search allele	609,394
	#13	#11 OR #12	1,129,551
	#12	Search polymorphisms	1,129,551

Domain(s)	Search	Query	Items found
	#11	Search polymorphism	1,129,551
	#10	#7 OR #8 OR #9	307,093
	#9	Search "single nucleotide polymorphism"	234,047
	#8	Search SNPs	102,922
	#7	Search SNP	171,697
	#6	#4 OR #5	974,644
	#5	Search genotypes	974,644
	#4	Search genotype	974,258
	#3	#1 OR #2	6,097,769
	#2	Search genes	6,096,771
	#1	Search gene	6,096,762

Table S2. Risk of bias assessment

No	Author	Year	Information bias			Confounding bias		Selective outcome report	HWE
			Ascertainment of case	Ascertainment of control	Ascertainment of genotyping examination	Population stratification	Other confounding bias		
1	Bai X, et al.	2018	🟢	🟢	🟡	🟢	🟢	🟢	🟢
2	Willrich, et al.	2018	🟢	🟢	🟡	🟢	🔴	🟢	🔴
3	Bakar, et al.	2017	🟢	🟢	🟡	🟢	🟢	🟢	🟢
4	Liu JE, et al.	2017	🟢	🟢	🟡	🟢	🟢	🟢	🟢
5	Khine H.,et al.	2016	🟢	🟢	🟡	🟢	🟢	🟢	🔴
6	Sai K, et al.	2016	🟢	🟢	🟡	🟢	🔴	🟢	🔴
7	Mirošević S, et al.	2015	🟢	🟢	🟡	🟢	🟢	🟢	🟢
8	Ferrari M, et al.	2014	🟢	🟢	🟡	🟢	🟢	🟢	🟢
9	Carr DF, et al.	2013	🟢	🟢	🟢	🟢	🟢	🟢	🟢
10	Brunham LR, et al.	2012	🟢	🟢	🟢	🟢	🟢	🟢	🔴
11	Donnelly LA, et al.	2011	🟢	🟢	🟢	🟢	🟢	🟢	🟢
12	Marciante KD, et al.	2011	🟢	🟢	🟢	🟢	🟢	🟢	🟢
13	Linde R, et al.	2010	🟢	🟢	🟡	🟡	🔴	🟢	🔴
14	Voora D, et al.	2009	🟢	🟢	🟢	🟢	🟢	🟢	🟢
15	Link E, et al.	2008	🟢	🟢	🟢	🟢	🟢	🟢	🟢

🔴 possible/high risk of bias 🟡 unclear or insufficient information to assess risk of bias 🟢 low/no risk of bias

Table S3. Allele frequencies of major and minor allele for *SLCO1B1* gene rs4149056 polymorphisms by ethnicity

study	author	Year	Myopathy					Control					C vs T		HWE
			No. of allele in case	T allele		C allele		No. of allele in control	T allele		C allele		OR	95%CI	P-value
				count	freq.	count	freq.		count	freq.	count	freq.			
Caucasian															
1	Willrich,et al.	2018	**178**	155	0.87	23	0.13	**178**	144	0.81	34	0.19	0.63	0.35, 1.12	**0.512**
2	Bakar,et al.	2017	**250**	201	0.80	49	0.20	**952**	818	0.86	134	0.14	1.49	1.04, 2.14	**0.568**
3	Mirošević Skvrce,et al.	2015	**120**	90	0.75	30	0.25	**180**	159	0.88	21	0.12	2.52	1.37, 4.67	**0.599**
4	Ferrari M,et al.	2014	**66**	29	0.44	37	0.56	**66**	49	0.74	17	0.26	3.68	1.76, 7.67	**1.00**
5	Carr DF,et al.	2013	**152**	110	0.72	42	0.28	**744**	621	0.83	123	0.17	1.93	1.29, 2.89	**0.709**
6	Brunham LR, et al.	2012	**50**	38	0.76	12	0.24	**166**	134	0.81	32	0.19	1.32	0.62, 2.81	**0.068**
7	Marciante KD,et al.	2011	**370**	281	0.76	89	0.24	**1464**	1260	0.86	204	0.14	1.95	1.47, 2.58	**0.95**
8	Donnelly LA, et al.	2011	**1632**	1357	0.83	275	0.17	**2550**	2158	0.85	392	0.15	1.12	0.94, 1.32	**0.085**

study	author	Year	Myopathy					Control					C vs T		HWE
			No. of allele in case	T allele		C allele		No. of allele in control	T allele		C allele		OR	95%CI	P-value
				count	freq.	count	freq.		count	freq.	count	freq.			
9	Linde R, et al.	2010	**54**	40	0.74	14	0.26	**38**	34	0.89	4	0.11	2.98	0.89, 9.89	**1.00**
10	Voora D, et al.	2009	**194**	155	0.80	39	0.20	**702**	610	0.87	92	0.13	1.67	1.10, 2.52	**0.482**
11	Link E, et al.	2008	**170**	93	0.55	77	0.45	**180**	157	0.87	23	0.13	5.65	3.32, 9.62	**0.147**
	**Pooled**			**0.74 (0.68, 0.79)**		**0.26 (0.21,0.32)**			**0.85 (0.84,0.86)**		**0.15 (0.14,0.16)**		**1.84**	**(1.35, 2.53)**	
Asian															
1	Sai K, et al.	2016	**104**	82	0.79	22	0.21	**5744**	4923	0.86	821	0.14	1.61	0.99, 2.59	**0.98**
2	Bai X, et al.	2018	**102**	74	0.73	28	0.27	**1402**	1192	0.85	210	0.149	2.15	1.36, 3.40	**0.000**
	**Pooled**			**0.76 (0.70,0.82)**		**0.24 (0.18 ,0.30)**			**0.86 (0.85,0.86)**		**0.14 (0.14,0.15)**		**1.87**	**(1.34, 2.60)**	
	Mixed ethnicity														
**1**	Khine H, et al.	2016	**194**	153	0.79	41	0.21	**362**	304	0.84	58	0.16	0.71	0.46, 1.11	**1.00**

study	author	Year	Myopathy					Control					C vs T		HWE
			No. of allele in case	T allele		C allele		No. of allele in control	T allele		C allele		OR	95%CI	P-value
				count	freq.	count	freq.		count	freq.	count	freq.			
	All ethnicity														
	**Pooled**				**0.75 (0.70 ,0.79)**		**0.25 (0.21,0.30)**			**0.85 (0.84, 0.86)**		**0.15 (0.14,0.16)**	**1.80**	**(1.40, 2.32)**	

Abbreviations: CI = confidence interval; freq.= frequency; HWE = Hardy-Weinberg Equilibrium; OR= Odds ratio

Table S4. Allele frequencies of major and minor allele for *SLCO1B1* gene rs2306283 polymorphisms by ethnicity

study	author	Year	Myopathy					Control					G vs A		HWE
			No. of allele in case	A allele		G allele		No. of allele in control	A allele		G allele		OR	95%CI	P value
				count	freq.	count	freq.		count	freq.	count	freq.			
Caucasian															
1	Willrich,et al.	2018	**178**	102	0.57	76	0.43	**178**	111	0.62	67	0.38	1.23	0.81, 1.89	**0.28**
2	Mirošević Skvrce,et al.	2015	**120**	65	0.54	55	0.46	**180**	112	0.62	68	0.38	1.39	0.87, 2.23	**0.33**
3	Ferrari M,et al.	2014	**66**	62	0.94	4	0.06	**66**	54	0.82	12	0.18	0.29	0.09, 0.95	**0.56**
4	Donnelly LA,et al.	2011	**1632**	1031	0.63	601	0.37	**2550**	1548	0.61	1002	0.39	0.90	0.79, 1.02	**0.89**
	**Pooled**			**0.67 (0.51,0.84)**		**0.33 (0.16,0.49)**			**0.66 (0.58,0.73)**		**0.34 (0.27,0.42)**		**1.00**	**(0.71, 1.43)**	
Asian															
1	Liu JE,et al.	2017	**296**	66	0.22	230	0.78	**510**	142	0.28	368	0.72	1.35	0.96, 1.88	**0.24**
**2**	Bai X, et al.	2018	**102**	25	0.25	77	0.75	**1402**	329	0.23	1073	0.77	0.94	0.59 1.51	0.46

study	author	Year	Myopathy					Control					G vs A		HWE
			No. of allele in case	A allele		G allele		No. of allele in control	A allele		G allele		OR	95%CI	P value
				count	freq.	count	freq.		count	freq.	count	freq.			
	Pooled				0.23 (0.19,0.27)		0.77 (0.73,0.81)			0.25 (0.23,0.26)		0.75 (0.74,0.77)	1.17	(0.84, 1.64)	
All ethnicity															
	Pooled				0.53 (0.32,0.73)		0.47 (0.27,0.68)			0.53 (0.34,0.71)		0.47 (0.29,0.66)	1.06	(0.83, 1.35)	

Abbreviations: CI = confidence interval; freq.= frequency; HWE = Hardy-Weinberg Equilibrium

Table S5. Pooled genotypic effects of *SLCO1B1* gene rs2306283 polymorphisms in Caucasian and Asian with statin users.

study	author	Year	Myopathy				Control				GG vs AA		AG vs AA	
			No. of subjects in case	Genotype			No. of subjects in control	Genotype			OR1	95%CI	OR2	95%CI
				AA	AG	GG		AA	AG	GG				
Caucasian														
1	Willrich,et al.	2018	**89**	28	46	15	**89**	37	37	15	1.32	0.55, 3.15	1.64	0.85, 3.16
2	Mirošević Skvrce,et al.	2015	**60**	18	29	13	**90**	37	38	15	1.78	0.70, 4.52	1.57	0.75, 3.30
3	Ferrari M,et al.	2014	**33**	29	4	0	**33**	21	12	0	0.73	0.01, 38.20	0.26	0.08, 0.88
4	Donnelly LA,et al.	2011	**816**	316	399	101	**1275**	471	606	198	0.76	0.58, 1.00	0.98	0.81, 1.19
	**Pooled**										**0.83**	**(0.64, 1.06)**	**1.00**	**(0.85, 1.20)**
Asian														
1	Bai X, et al.	2018	**51**	4	17	30	**701**	42	245	414	0.76	0.26, 2.26	0.73	0.23, 2.27
2	Liu JE,et al.	2017	**148**	10	46	92	**255**	16	110	129	1.14	0.50, 2.63	0.67	0.28, 1.58
	**Pooled**										**0.98**	**(0.50, 1.96)**	**0.68**	**(0.34, 1.36)**

Table S6. Exploring the source of heterogeneity and subgroup analysis of *SLCO1B1* gene rs4149056 polymorphisms in Caucasians

	No. of studies	OR_1 (CC vs TT)			OR_2 (TC vs TT)		
		OR (95% CI)	P value	I^2 (%)	OR (95% CI)	P value	I^2 (%)
**- Caucasian Overall**	**10**	**2.92 (1.59, 5.34)**	**0.013**	**56.9**	**1.61 (1.20, 2.16)**	<0.001	**72.5**
**Source of heterogeneity**							
Mean age	10		0.674	60.24		0.943	75.04
Percent of female	10		0.905	59.95		0.588	74.08
Duration of therapy	5		0.373	62.88		0.327	74.40
Statin type	12		0.910	52.44		0.420	70.54
**Subgroup by statin type**							
simvastatin	5	2.81 (1.17, 6.77)	0.008	70.8	1.78 (1.15, 2.77)	<0.001	82.6
atorvastatin	3	3.95 (1.23, 12.63)	0.356	3.3	1.98 (1.11, 3.52)	0.716	0.0

Table S7. Egger's tests for *SLCO1B1* gene rs4149056 polymorphisms in Caucasians

	No. of sub-studies	OR_1 (CC vs TT)			OR_2 (TC vs TT)		
		**Coef. of Egger's test**	**SE**	**P-value**	**Coef. of Egger's test**	**SE**	**P-value**
Caucasian	10	-0.76	1.03	0.481	1.97	0.94	0.069

Figure S1. Funnel plots of publication bias of *SLCO1B1* gene rs4149056 polymorphisms in Caucasian with statin users.

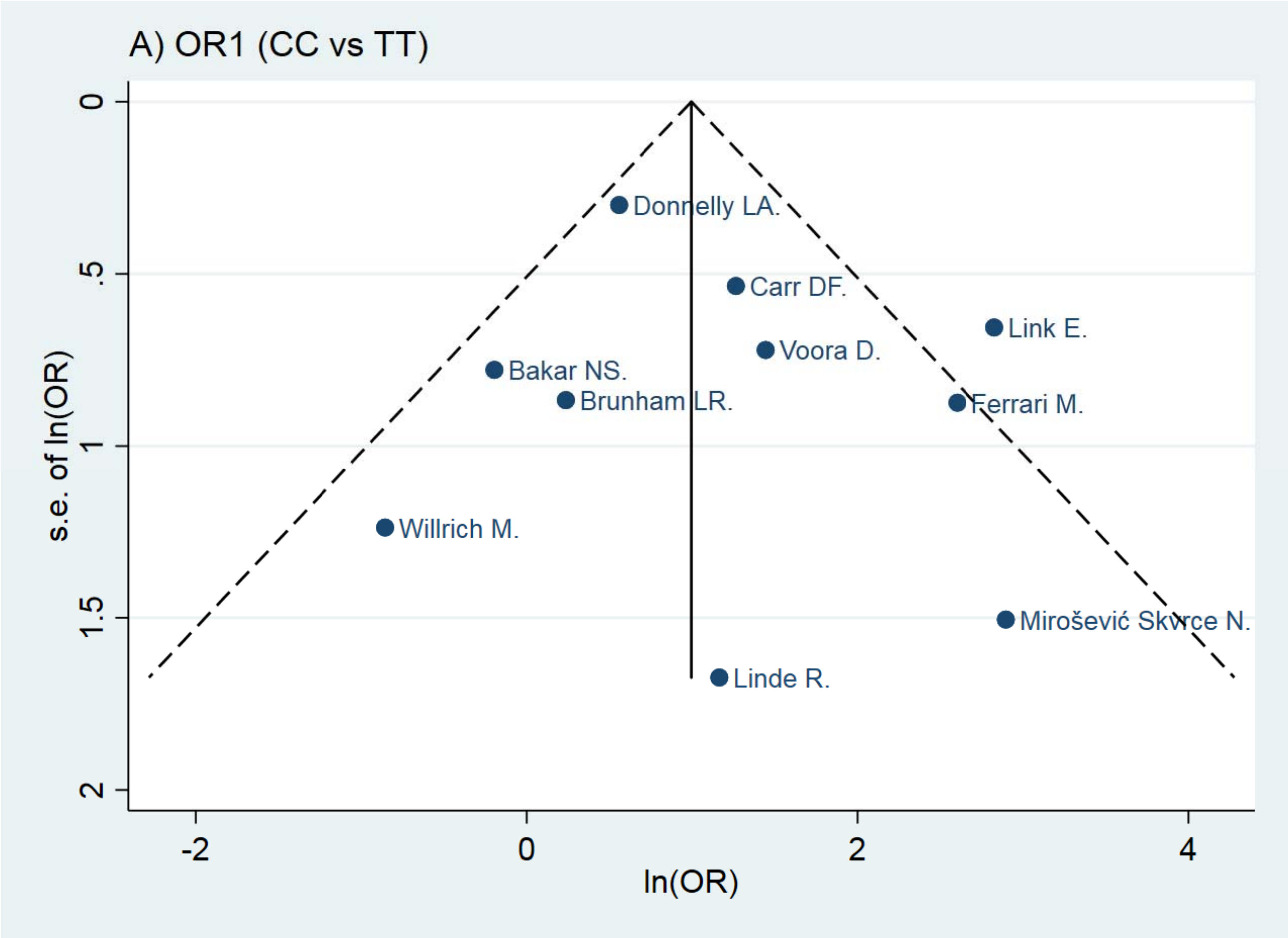

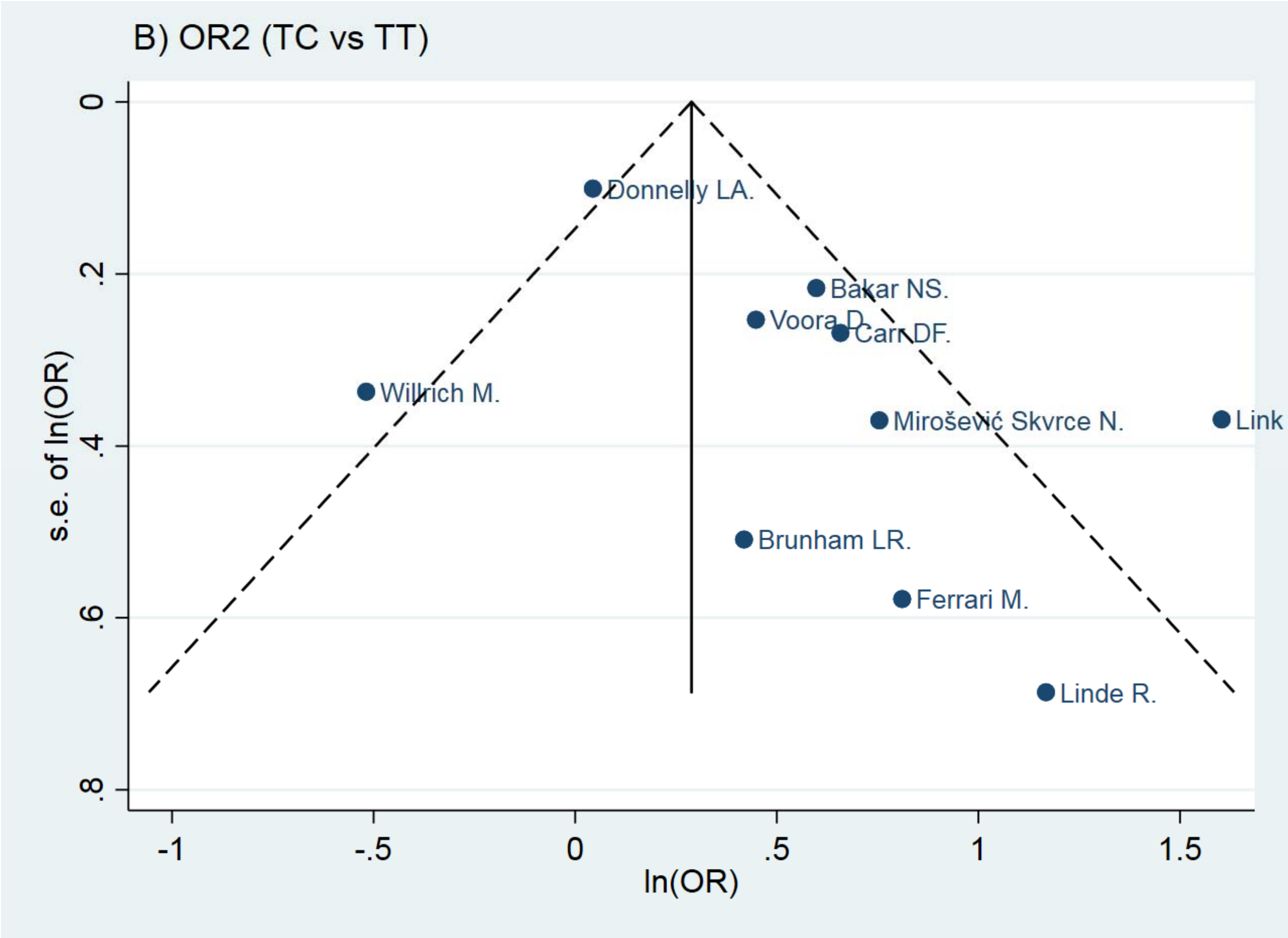
B) OR2 (TC vs TT)
s.e. of ln(OR)
0
.2
.4
.6
.8
-1
-.5
0
.5
1
1.5
ln(OR)
Donnelly LA.
Bakar NS.
Voora D.
Carr DF.
Willrich M.
Mirošević Skvrce N.
Link
Brunham LR.
Ferrari M.
Linde R.

Figure S2. Funnel plots of publication bias of *SLCO1B1* gene rs4149056 polymorphisms in Caucasian with atorvasstatin users.

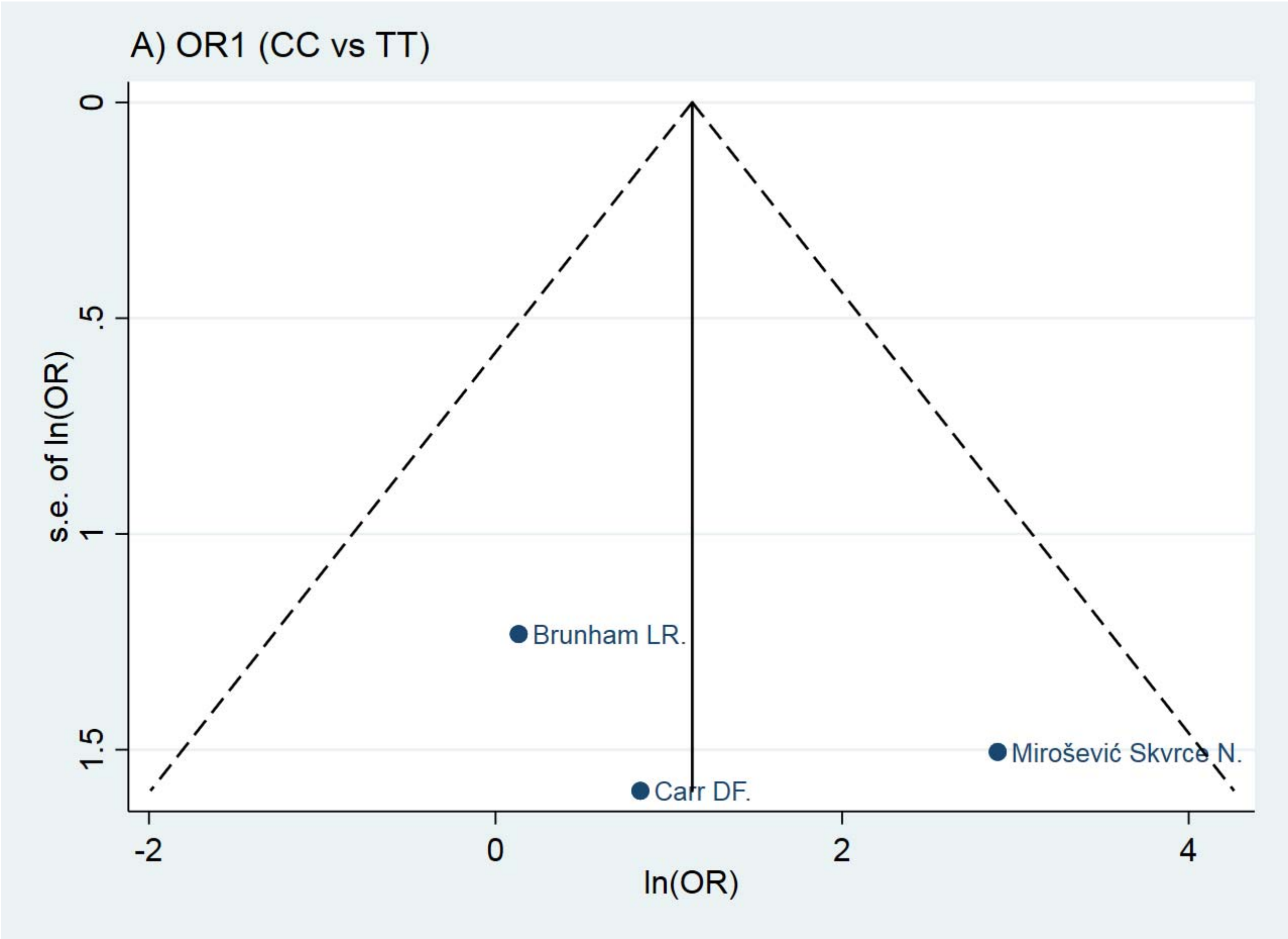

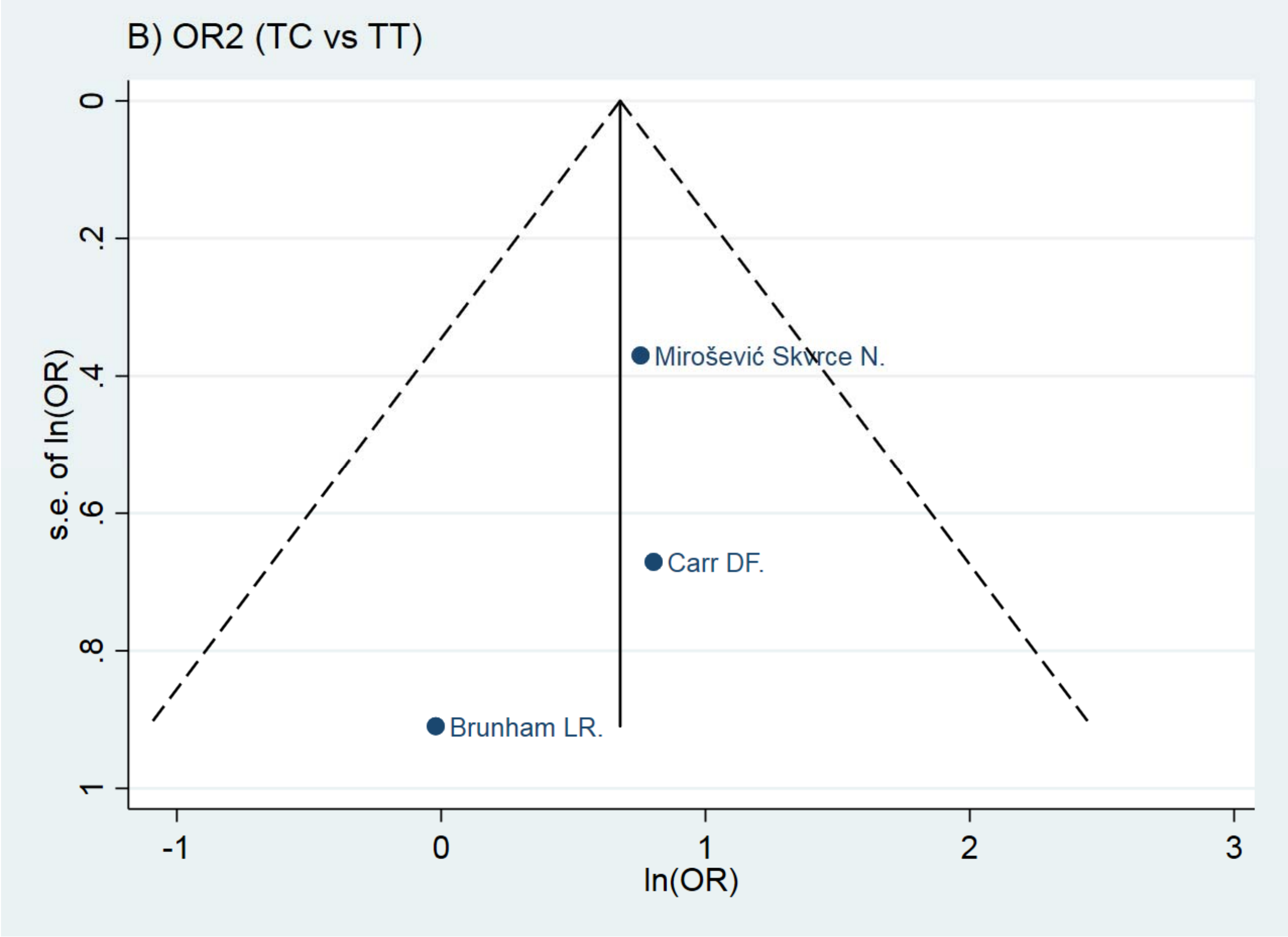
B) OR2 (TC vs TT)
0
.2
.4
.6
.8
1
s.e. of ln(OR)
Mirošević Skvrce N.
Carr DF.
Brunham LR.
-1
0
1
2
3
ln(OR)

Figure S3. Funnel plots of publication bias of *SLCO1B1* gene rs4149056 polymorphisms in Caucasian with simvastatin users.

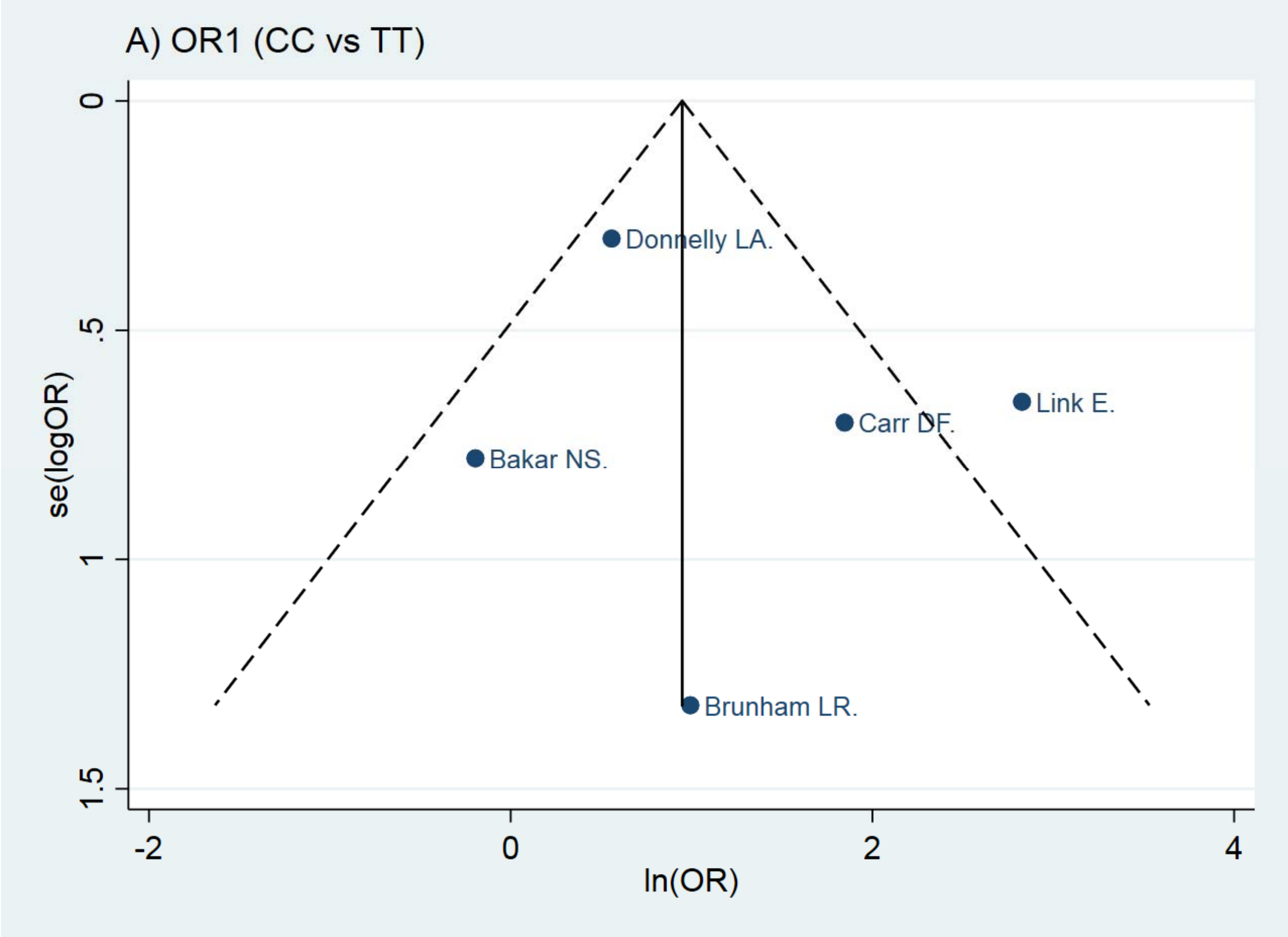

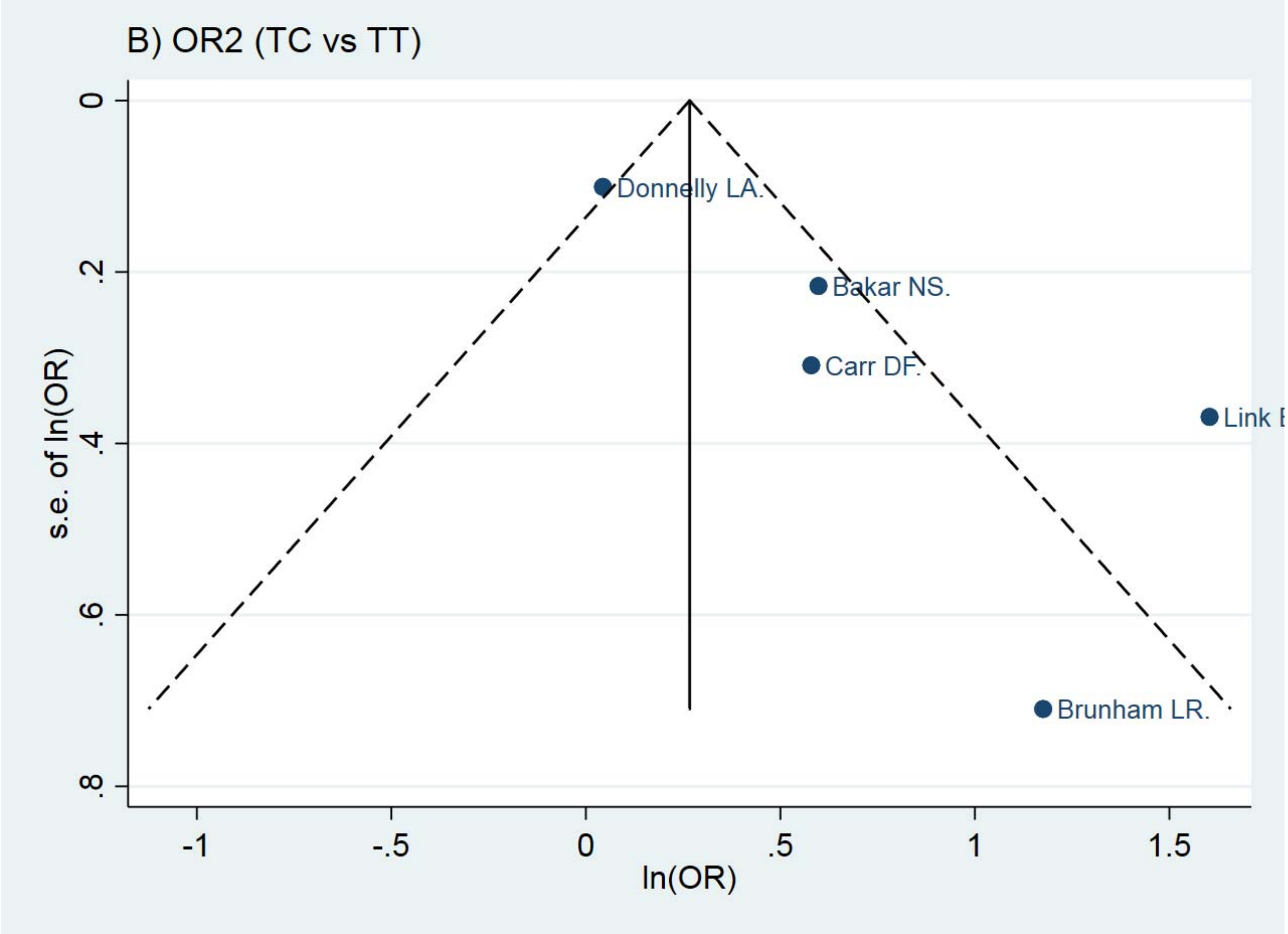

B) OR2 (TC vs TT)
0
.2
.4
.6
.8
s.e. of ln(OR)
Donnelly LA.
Bakar NS.
Carr DF.
Link E
Brunham LR.
-1
-.5
0
.5
1
1.5
ln(OR)

Figure S4. Funnel plots of publication bias of *SLCO1B1* gene rs2306283 polymorphisms in Caucasian with statin users.

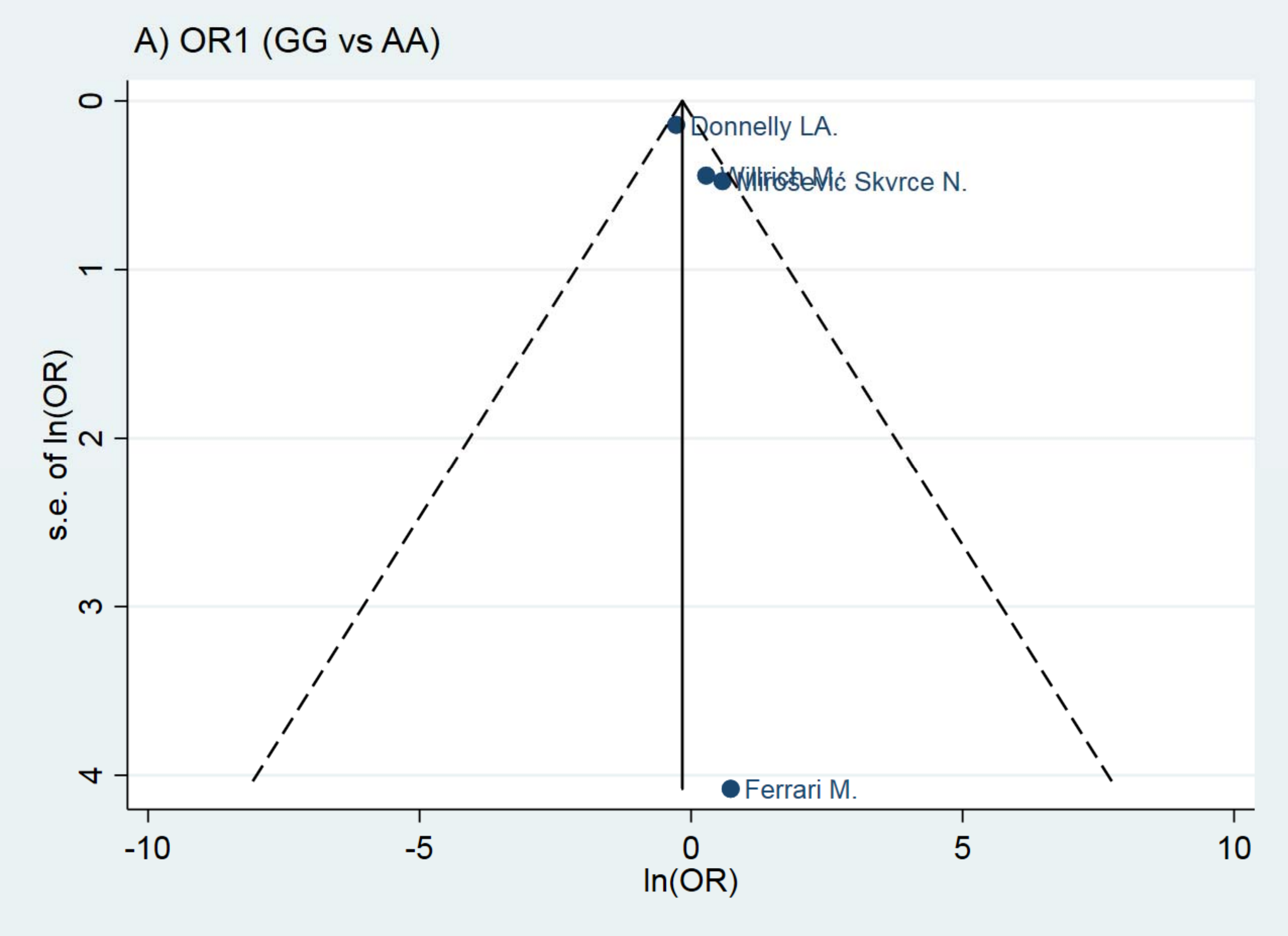

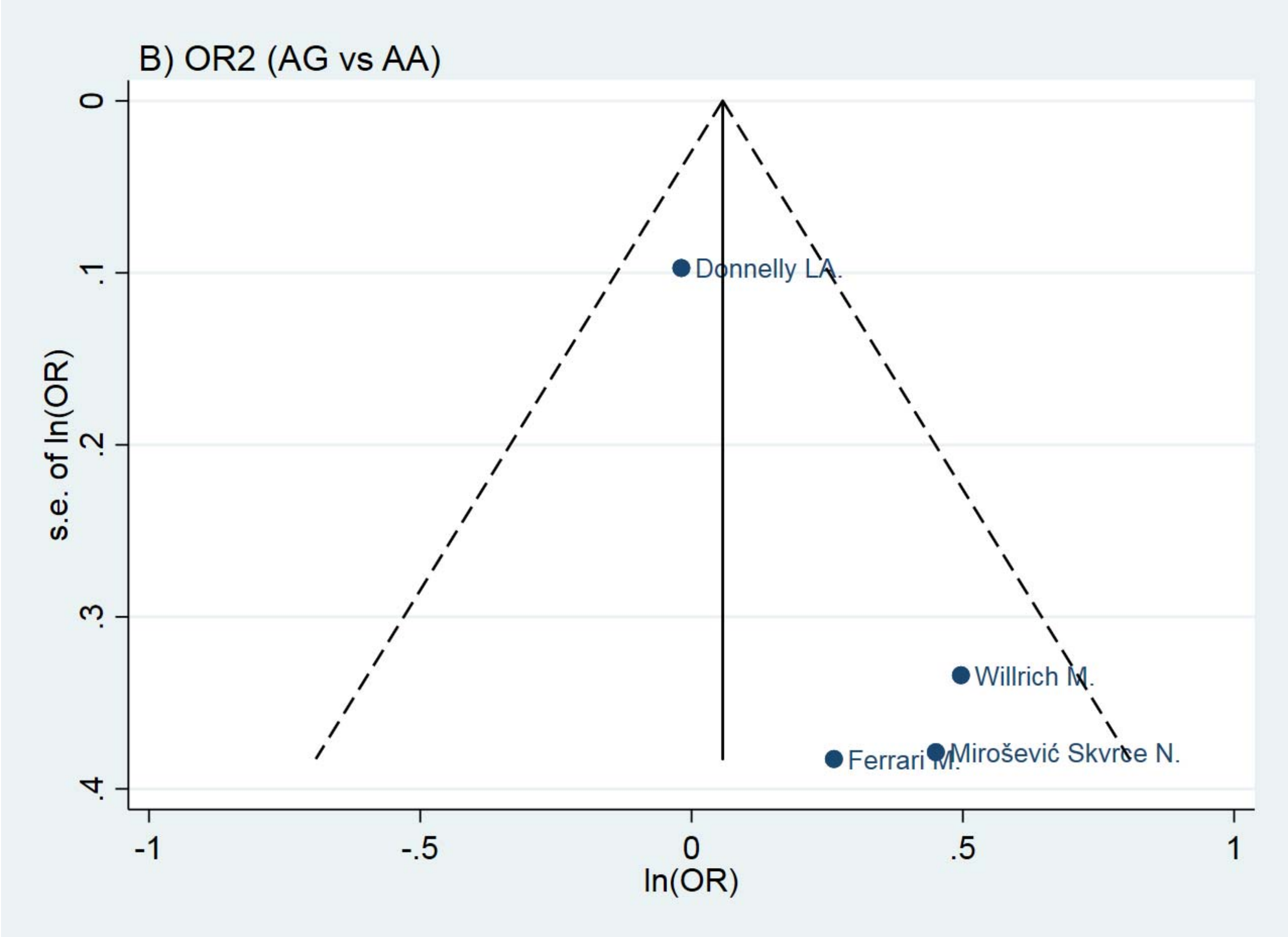
B) OR2 (AG vs AA)
s.e. of ln(OR)
0
.1
.2
.3
.4
Donnelly LA.
Willrich M.
Ferrari M.
Mirošević Skvrce N.
-1
-.5
0
.5
1
ln(OR)

Figure S5. Funnel plots of publication bias of *SLCO1B1* gene rs2306283 polymorphisms in Asian with statin users.

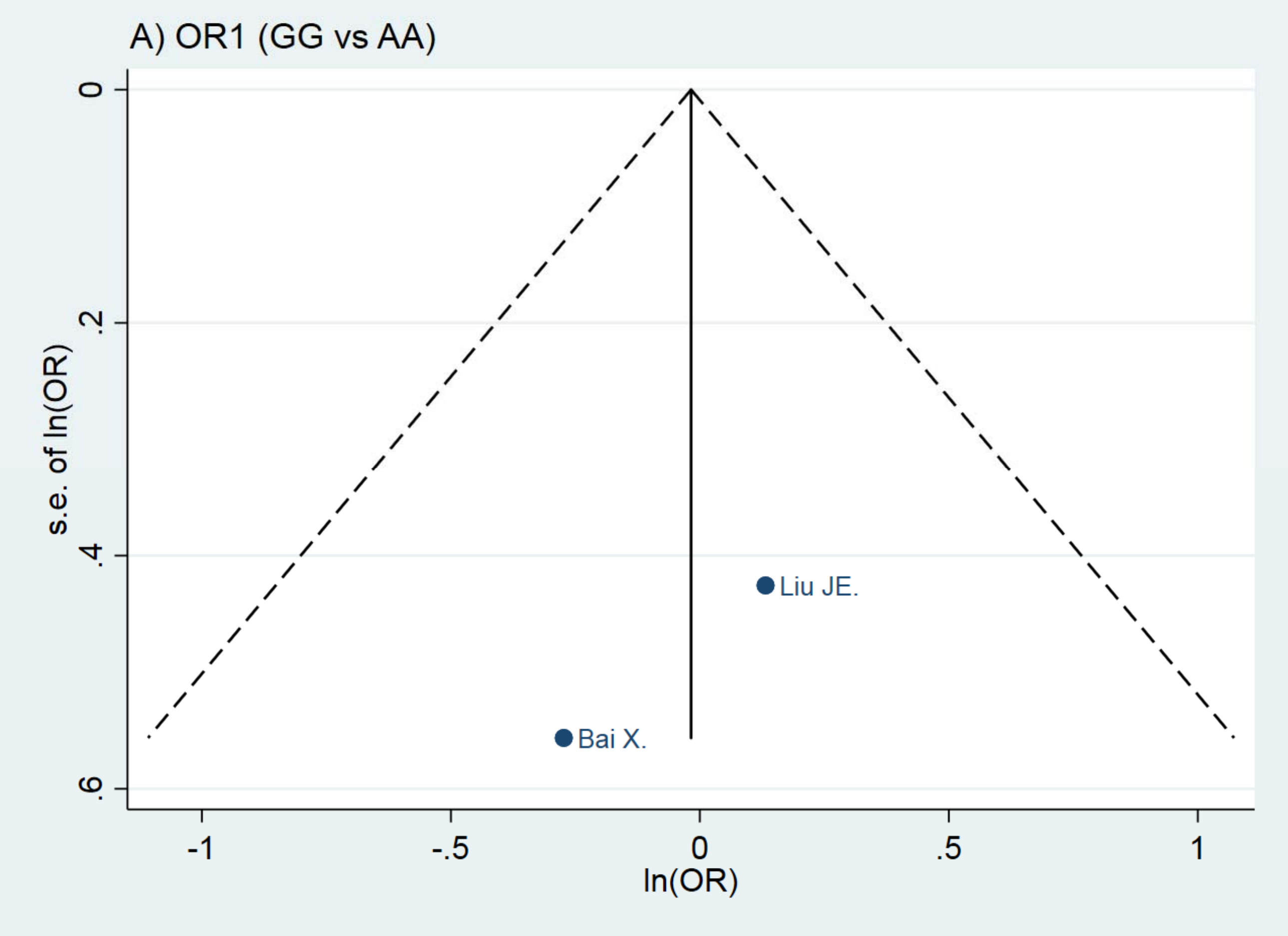

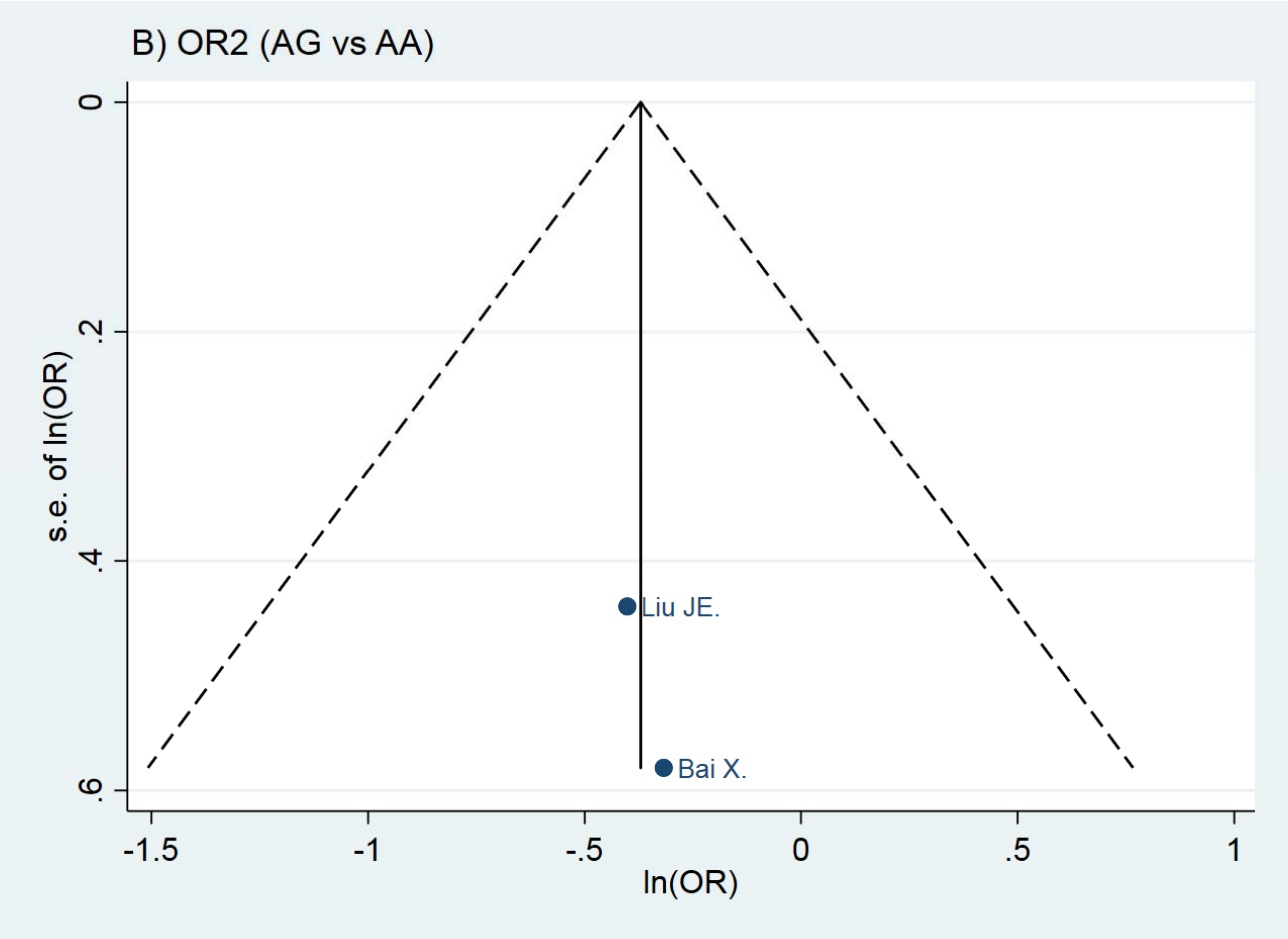
B) OR2 (AG vs AA)
s.e. of ln(OR)
0
.2
.4
.6
Liu JE.
Bai X.
-1.5
-1
-.5
0
.5
1
ln(OR)
